# Spontaneously Low Protein Intake in Elderly CKD Patients: Myth or Reality? Analysis of Baseline Protein Intake in a Large Cohort of Patients with Advanced CKD

**DOI:** 10.3390/nu13124371

**Published:** 2021-12-06

**Authors:** Massimo Torreggiani, Antioco Fois, Maria Rita Moio, Antoine Chatrenet, Béatrice Mazé, Françoise Lippi, Jerome Vigreux, Coralie Beaumont, Giulia Santagati, Noemi Paulin, Giorgina Barbara Piccoli

**Affiliations:** Néphrologie et Dialyse, Centre Hospitalier Le Mans, 194 Avenue Rubillard, 72037 Le Mans, France; maxtorreggiani@hotmail.com (M.T.); afois@ch-lemans.fr (A.F.); mariaritamoio@gmail.com (M.R.M.); achatrenet@ch-lemans.fr (A.C.); bmaze@ch-lemans.fr (B.M.); flippi@ch-lemans.fr (F.L.); jvigreux@ch-lemans.fr (J.V.); cbeaumont@ch-lemans.fr (C.B.); giulia.santagati@hotmail.it (G.S.); noemypaulin@gmail.com (N.P.)

**Keywords:** low-protein diet, CKD, elderly, protein intake, survival

## Abstract

The recent guidelines on nutritional management of chronic kidney disease (CKD) advise a reduction in protein intake as early as CKD stage 3, regardless of age, to slow kidney function impairment. However, since elderly patients are usually considered as having a spontaneously reduced protein intake, nutritional interventions to reduce protein intake are often considered futile. This study aimed to assess the baseline protein intake of elderly CKD patients referred for nephrology care, and explore the need for dietary evaluations, focusing on the current recommendations for protein restriction in CKD. This is an observational study of CKD patients followed in the unit dedicated to advanced CKD patients in Le Mans, France. Patients with stages 3 to 5 not on dialysis were included. All patients were evaluated by an expert dietician to assess their baseline protein intake, whenever possible on the basis of a 7-days diet journal; when this was not available, dietary recall or analysis of delivered meals was employed. Demographic characteristics, underlying kidney disease, Charlson Comorbidity Index (CCI), Malnutrition-Inflammation Score (MIS), Subjective Global Assessment (SGA) and clinical and laboratory data were recorded. Between 15 November 2017 and 31 December 2020, 436 patients were evaluated in the unit. Their age distribution was as follows: “young”: <60 (*n* = 62), “young-old”: 60–69 (*n* = 74), “old”: 70–79 (*n* = 108), “old-old”: 80–89 (*n* = 140) and “oldest-old”: ≥90 (*n* = 54). The prevalence of vascular nephropathies was higher in patients older than 70 years compared to younger ones, as did CCI and MIS (*p* < 0.001). Moderate nutritional impairment (SGA: B) was higher in elderly patients, reaching 53.7% at ≥90, while less than 3% of patients in the overall cohort were classified as SGA C (*p* < 0.001). The median protein intake was higher than the recommended one of 0.8 g/kg/day in all age groups; it was 1.2 g/kg/day in younger patients and 1.0 thereafter (*p* < 0.001). Patient survival depended significantly on age (*p* < 0.001) but not on baseline protein intake (*p* = 0.63), and younger patients were more likely to start dialysis during follow-up (*p* < 0.001). Over half of the patients, including the old-old and oldest-old, were still on follow-up two years after referral and it was found that survival was only significantly associated with age and comorbidity and was not affected by baseline protein intake. Our study shows that most elderly patients, including old-old and extremely old CKD patients, are spontaneously on diets whose protein content is higher than recommended, and indicates there is a need for nutritional care for this population.

## 1. Introduction

The burden of chronic kidney disease (CKD), whose prevalence is estimated to be as high as 8 to 15% of the world’s population, peaks in the elderly and is frequently associated with high comorbidity. This burden is considerable and its prevalence is challenging almost all health care systems [1,2,3]. While undeniable advances in the care of glomerular diseases and of some hereditary diseases, including autosomal dominant polycystic kidney disease (ADPKD), have delayed the need for renal replacement therapy, the increasing number of elderly patients and of patients with diabetes, hypertension, and diffuse vascular disease points to a need for optimizing comprehensive care [1,4]. Multidisciplinary management and the integration of pharmacological and non-pharmacological interventions, including physical activity and nutritional management, play a vital role in avoiding or delaying the need for renal replacement therapy.

The recent KDOQI guidelines on nutritional management of CKD patients widened the spectrum of nutritional interventions, in particular of protein restriction, to CKD stage 3, regardless of age, underlining that optimization of protein intake is associated with decreased mortality and morbidity [5].

However, it is commonly held that elderly patients spontaneously decrease their protein intake [6,7,8,9]. Spontaneous protein intake tends also to decrease in CKD patients [6,7,8]. In clinical practice, the combination of these two observations leads quite often to a therapeutic minimalism, and nutritional interventions aimed at reducing protein restriction in elderly CKD patients are not widely practiced [10]. This attitude is also enhanced by the consideration elderly patients are considered as to be reluctant to change their dietary habits [6]. While data is lacking on the diffusion of the prescription of nutritional interventions aimed at controlling protein intake, in particular in the predominant elderly CKD population, the team involved in the present survey experienced these barriers, being the first one to prescribe nutritional interventions in elderly CKD patients in the area in Central France where the present study was conducted [11].

Of note, the recent KDOQI guidelines are in partial contrast with the position of geriatric societies, and, for example, the recent ESPEN guidelines suggest that high protein diets may serve to counterbalance sarcopenia and malnutrition in the elderly [5,12]. Filling the current gap of knowledge on what is the actual protein intake in different populations of elderly CKD patients referred to nephrology care may help understand the actual need for nutritional management, and help to better define the clinical and educational needs tailored to this fragile population.

There is no univocal definition of “elderly patients”, as definitions have changed over time, to some extent because of the prolonged life-span of the population, in particular in high-income countries [13,14,15,16,17]. Different cut-points for this definition are chosen ranging from over 60 or 65 years, and eventually graded into “young-old” (usually defined as 60–70 years of age), to “oldest-old” (usually defined as over 80 or 90 years of age) [13,14,15,16,17].

Regardless of the specific definition, very few studies have sought to quantify the burden of elderly patients in a nephrology outpatient clinic, and, this is expected to vary widely, not only in terms of the prevalence of CKD in various populations, but also in line with referral criteria, which are often minimalist for the oldest age groups [4]. Having a better definition of the elderly CKD population and of their nutritional habits is therefore crucial to planning targeted actions, in particular dietary interventions.

In this study, we aimed to assess the nutritional status and dietary habits, with regard to protein intake, of elderly CKD patients referred to a dedicated large outpatient unit (UIRAV: Unitè pour la prise en charge de l’Insuffisance Rénale AVancée), in a large public hospital in Central France.

## 2. Materials and Methods

### 2.1. Setting of Study

The present study was undertaken at Centre Hospitalier Le Mans (CHM), one of the largest non-university hospitals in France. CHM has a nephrology service with a network of outpatient care facilities (consultations and day-hospital) and is the only hospital in the Department of Sarthe with nephrology beds (Sarthe: 562,177 inhabitants on 1 January 2021) [18]. The hospital is situated in the main city of the department, Le Mans, which counted 146,090 inhabitants on the same date [19].

### 2.2. The Unit Dedicated to Advanced CKD (UIRAV)

Care at UIRAV in Le Mans is delivered by two senior nephrologists, four dieticians, one or two residents and a small group of nurses. Patients are followed-up with outpatient visits or in day hospital, if they need intravenous drug treatment or complex diagnostic assessments; hospitalization is electively performed in the nephrology ward. While the unit also serves fragile patients with lesser degrees of kidney function impairment and is the setting of follow-up of high-risk pregnancies, only patients with CKD stages 3–5 not on dialysis were selected for the present analysis.

### 2.3. Dietary Assessment

As a rule, the assessment of energy and protein intake was based on a 7-day food diary, integrated with a detailed interview with the dietitian. Through the interview, all the records are reviewed and, when necessary, integrated. Furthermore, the serving size is controlled with a visual aid (Supplementary Figure S1).

If no diary is available (non-adherence, cognitive impairment) the energy and protein intake is evaluated either based on the patient’s dietary recall (3 days) or, in the case of patients who depend upon meal delivery or reside in a retirement home, the caregivers and the responsible for meal delivery are contacted.

The presence of a family member is welcomed in all evaluations, in particular in elderly patients and in patients with cognitive impairment.

The duration of the first nutritional assessment is scheduled in one hour, and the subsequent evaluations last, on average, 30 min.

The dietitian team is composed by 4 dietitians, who gained specific experience in kidney diseases. The nephrologists of the unit have specific experience on nutritional management of kidney diseases, and a periodic (at least every 2 months) meeting is organized for updates, and to discuss specific cases, with the aim of granting a homogeneous approach to evaluation and counseling.

For patients able to correctly perform a 24-h urine collection, analysis of 24-h urinary urea is also used to assess protein intake, employing the Maroni-Mitch formula [20]; the results are considered as a coherence control, to confirm the results of the diet journal.

In the absence of a universally agreed formula for the definition of ideal body weight, protein intake was assessed per kilogram of real body weight, and an average between real and ideal body weight was used only for patients whose body mass index (BMI) was >40 kg/m^2^.

#### Definition of Nutritional Status and Comorbidities

Nutritional status was evaluated by means of the Malnutrition-Inflammation Score (MIS, scale: 0–30), and a Subjective Global Assessment (SGA: A, B or C) by the nephrologists in the unit [21,22], using the Charlson Comorbidity Index (CCI, scale: 0–33) to assess comorbidity [23].

All patients were examined at baseline and followed-up to identify signs of protein-energy wasting (PEW), such as reduction in body weight (unwanted and unexplained by oedema reduction), reduction in lean body mass (clinical assessment, or, when necessary, bioimpedance), reduction in serum albumin, prealbumin or total proteins, vitamin deficits or unexplained anemia, in the absence of acute or chronic inflammation, or other clinical markers of poor nutrition.

Once on follow-up, consultation with a dietitian, including a review of the dietary journal kept by the patient, was scheduled at least twice a year.

### 2.4. Dietary Management

At UIRAV, when the daily protein intake is higher than the recommended one (0.8 per kg of ideal body weight), in the absence of contraindications, including protein wasting, low-energy intake or being in the recovery phase from an acute disease, the nephrologist prescribes normalization or a reduction in protein intake, based on baseline intake, nutritional status, trajectory of CKD progression, proteinuria, age, comorbidity and life expectancy. The dietary options are extensively discussed with the patient and, whenever possible, with caregivers, and the main nutritional strategy (mixed proteins or a plant-based diet) is agreed on [11,24].

Dialysis start is decided with an “intent to delay” policy, based on the usual clinical and biochemical markers of blood pressure control, fluid overload, hyperparathyroidism or any clinical element suggesting uremic toxicity (anorexia, weight loss, nausea, malnutrition, restless leg syndrome). Whenever possible, dialysis is started in an incremental way.

### 2.5. Data Gathered

The following data were gathered: demographic (gender, age, country of origin), type of kidney disease; protein intake; MIS, SGA, CCI.

Clinical data included height, weight, BMI, blood pressure; laboratory data included urea, creatinine, electrolytes, albumin, total serum proteins, hemoglobin, parathyroid hormone, complete blood count, ferritin and transferrin. Data not shown in tables but recorded in the database, are available on request. Estimated glomerular filtration rate (eGFR) was assessed using the MDRD short and the CKD Epidemiology Collaboration (CKD-EPI) formulas [25,26].

### 2.6. Statistical Analysis

We defined “elderly patients” as aged over 60, and graded them into “young” (<60 years of age), “young-old” (60–69 years of age), “old” (70–79 years of age), “old-old” and “oldest-old” (80–90 and over 90 years of age, respectively).

To allow for contextualization with the local dietary habits, data on younger age groups were also gathered and analyzed.

The descriptive analysis was undertaken by decades of age at referral, while the smaller cohorts of patients aged less than 60 were merged. Survival analysis was performed by larger age groups (<60; 60–79; and ≥80 years) to allow having a sufficient numerosity for further stratification for potentially relevant variables, including level of protein intake at referral.

Statistical analyses were performed using JASP v0.14.1 (JASP Team 2020, Amsterdam University, The Netherlands) and RStudio v1.4.1 (R Core Team 2021, Vienna, Austria). Quantitative data were expressed as a median (min–max) and qualitative data were presented as proportions and percentages.

The normality and homoscedasticity hypotheses were tested with the Shapiro-Wilk and Levene’s tests, respectively, for continuous series. In cases that involved acceptance of a null hypothesis, the Student *t*-test was performed to compare two unpaired groups; otherwise the Wilcoxon rank sum test was used. Analysis of variance (ANOVA) was applied for additional group comparisons, or the Kruskal–Wallis test was performed. Proportions were tested using the Chi-square test, or the Fisher exact test in cases of small subsample cohorts (<5).

Survival, renal survival and total drop-out (death, dialysis start or loss to follow-up) was visualized by means of Kaplan-Meier plots, while groups (i.e., protein intake and age groups) were compared with the Log-rank test. In addition, the risk of death was tested through two Cox models with the following variables: (1) Protein intake at referral, dichotomized at ≥0.8 g/kg/day; (2) CCI ≥ the median (i.e., 8) in model 1 or age ≥ the median (i.e., 78) in models 2 and 3; female/male.

The two-sided alpha risk was set at 5%.

### 2.7. Ethical Issues

The study was conducted in accordance with the Declaration of Helsinki. The observational study, entitled “Interest and feasibility of a personalized dietary regimen in pre-dialysis patients” (Intérêt et faisabilité d’un régime adapté en pré-dialyse) involving all patients who attended at least one consultation at UIRAV in 2017–2020 was approved by the hospital’s ethics committee on 14 June 2018. Because of its retrospective, non-interventional nature, the study was approved by the Ethics Committee of the Centre Hospitalier du Mans with a positive deliberation without a protocol number (avis favourable, séance du 14 June 2018).

## 3. Results

### 3.1. General Baseline Characteristics

The main demographic characteristics of the 436 patients referred to the unit dedicated to advanced CKD (UIRAV), between the start of its activity (13 November 2017) and 31 December 2020 are reported in Table 1. The distribution of CKD stages was similar in all subsets of elderly patients, in keeping with a superimposable median e-GFR.

As expected, the prevalence of vascular nephropathies was higher in patients aged 70 or older than in younger patients and, in keeping with this diagnosis, most of the elderly patients did not display relevant proteinuria.

### 3.2. Comorbidity, Malnutrition-Inflammation Score and Subjective Global Assessment

Table 2 and Figures S2–S4 depict the distribution of the comorbidity burden, and describe the nutritional and comprehensive indexes employed in the definition of CKD patients.

The Charlson Comorbidity Index (CCI) was higher in patients older than 60 years compared to younger ones, partly due to the effect of age per se. The prevalence of diabetes was highest in the age groups 60–69 and 70–79, and decreased thereafter, suggesting a role of competitive mortality, while the prevalence of neoplastic diseases peaked at 80–89 years of age. Ischemic heart disease was present in about 40% of the patients over 50, underlining the close relationship between chronic kidney disease and cardiac impairment.

Subjective Global Assessment (SGA) defined our population as being generally well nourished, with only a small minority whose nutritional status was severely impaired (less than 2% in the overall cohort) (Supplementary Figure S4). Conversely, the prevalence of moderate nutritional impairment was higher in patients older than 60 years compared to younger ones, reaching 53.7% at 90 or older. It should be noted that the definition was not “age-adjusted” and may reflect age-related sarcopenia, more than the effect of CKD. In keeping with this observation, the median BMI was in the overweight range at all ages but decreased from about 29 kg/m^2^ in the age group 50–79 to 25.7 kg/m^2^ in patients ≥ 90 years old. The Malnutrition-Inflammation Score (MIS) was also higher in patients older than 60 years compared to younger ones, as graphically plotted in Supplementary Figure S3.

### 3.3. Biochemical Profile across Age Groups

Supplementary Table S1 summarizes the biochemical profile of different age groups in patients followed–up at UIRAV. Note that median cholesterol and albumin were in the normal range at all ages, and only a minority of cases had low albumin or low hemoglobin levels at referral. The distribution of the same biochemical markers across ages and stages in elderly patients is summarized in Supplementary Tables S2–S5.

### 3.4. Protein Intake in Different Age Groups

The median protein intake, assessed at baseline, was higher than the recommended 0.8 g/kg of body weight per day in all age groups. However, the median baseline protein intake decreased from 1.2 g/kg of body weight per day in patients under 60, to 1.1 g/kg/day at age 60–69 and to 1.0 thereafter. The prevalence of cases with normal or spontaneously reduced protein intake significantly increased in patients older than 60 years compared to younger ones (Table 3).

The distribution of protein intake across stages and ages is shown in Supplementary Table S6. It was found that the influence of stages is not significant in any age group, while, as also reported in Table 3, protein intake tends to decrease with age. Similar results were observed when sorting patients according to the Charlson Comorbidity Index, as reported in Supplementary Table S7.

### 3.5. Outcome Analysis: Survival, Renal Survival, Total Drop-Out

**Figure 1:**
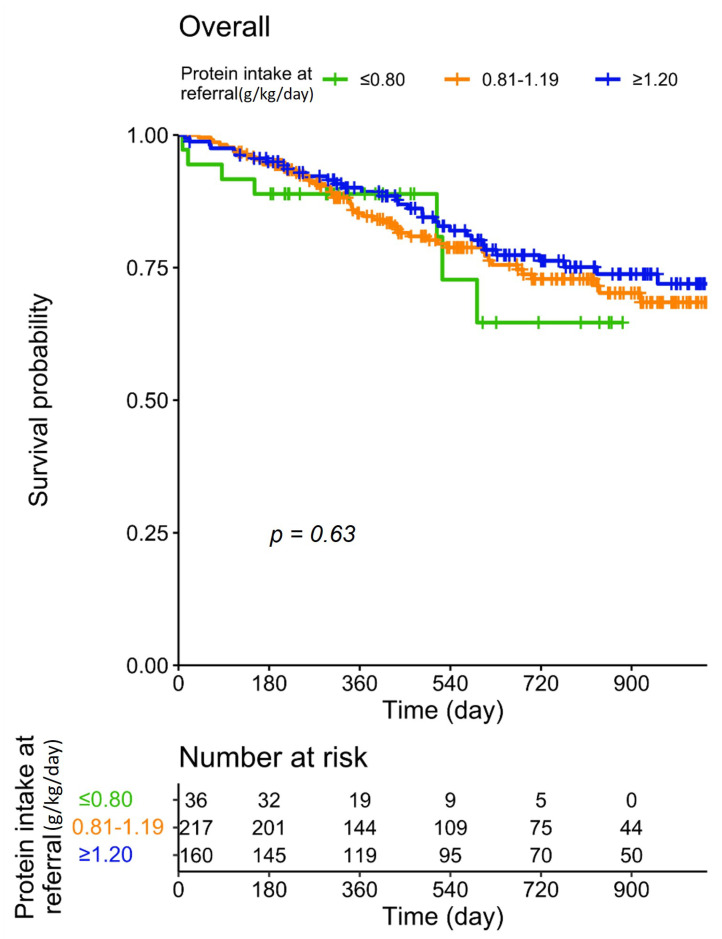
Patient survival according to protein intake at baseline.

Patient survival according to dietary protein intake at baseline is reported in Figure 1. It was found that differences in baseline protein intake did not significantly affect patient survival; in particular, no disadvantage was recorded in patients with a spontaneously lower protein intake.

**Figure 2:**
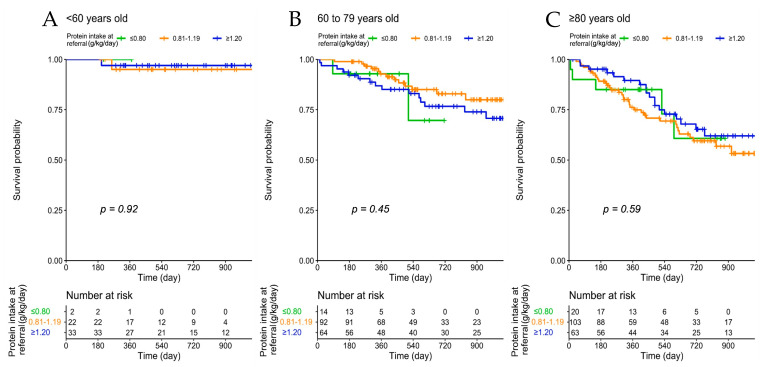

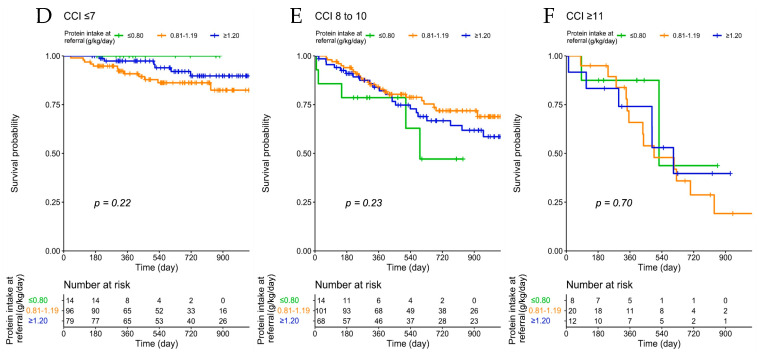
Patient survival according to protein intake at baseline, across age groups (**A**–**C**) and Charlson Comorbidity Index (**D**–**F**).

The analysis stratified according to age groups confirms the lack of a significant association between protein intake and mortality in all age groups and in groups defined according to CCI (Figure 2).

Renal survival and the total drop-out curve which calculates the percentage of patients that have died, started dialysis or been lost to follow-up, showing the “persistence” of patients in the setting of care, are reported in Supplementary Figures S5 and S6, respectively.

Notwithstanding the attrition caused by mortality and dialysis start, over half of the patients, including the old-old and oldest-old cases were still on follow-up two years after referral (Supplementary Figure S6). The overall survival of patients followed in our unit according to age is depicted in Supplementary Figure S7.

The lack of influence of a spontaneously lower protein intake is also confirmed by Cox analysis (Table 4 and Table 5, with protein intake dichotomized at ≥0.8 g/kg/day, and Supplementary Tables S8 and S9, with protein intake dichotomized at ≥1 g/kg/day).

## 4. Discussion

The present study was undertaken to contribute to filling the knowledge gap regarding the current dietary habits in elderly CKD patients referred for nephrology care; these data are precious to tailor nutritional approaches and educational interventions, and thereby contribute to answering open questions regarding the need for nutritional care, with specific regard to protein intake, in elderly CKD patients.

The main finding of this study is that, in a large cohort of CKD patients followed by a nephrology unit dedicated to the care of advanced CKD in central France, at referral most of the elderly patients, even the oldest ones, had a protein intake higher than the current recommendations. These data differ from other reports that suggest that most individuals normalize or reduce their protein intake as they age [8,27]. Furthermore, differently from previous reports, in the setting of study, protein intake did not decrease across stages and was similar in patients at higher or lower comorbidity (Supplementary Tables S6 and S7) [6,7,8].

In our setting, in Central France, where overweight and obesity are prevalent, and high protein intake is found in all age groups, our data, in keeping with the current literature [12], show that the main nutritional markers are different in different age groups, and the prevalence of SGA A (well nourished) is higher in the older age groups. While very few patients were categorized as SGA C, the prevalence of moderate signs of malnutrition is likewise higher in the “old-old” and “extremely old” patients, a finding that is in keeping with previous studies [28]. The same tendency was observed for BMI, while the prevalence of low albumin levels did not change among age groups (Table 2 and Table 3).

The prevalence of patients on high-protein diets, defined as greater or equal to 1.2 g/kg/day, progressively decreased from about 70% in younger patients to about 35% of patients aged over 80. Interestingly, about 20% of patients aged over 90 that had been referred for nephrology care had a protein intake ≥1.2 g/kg/day at their first assessment. While the prevalence of patients on a spontaneously normal (0.8 g/kg/day) or reduced protein intake was about 40% in the oldest-old, at least 60% of the patients aged ≥90, and the vast majority of old-old patients were theoretically amenable to reducing protein intake to stabilize or slow the progression of CKD (Table 3) [5].

There are several potential explanations for the differences between our findings and those of other studies in the literature, reporting on reduction of protein intake across ages and in more advanced CKD stages [6,7,8]. One could be linked to the high number of overweight and obese individuals in our cohort of CKD patients, whose median BMI was in the overweight range across all ages, including the oldest-old. This partially reflects the prevalence of obesity in the setting of study, a rural area in Central France [29,30]. A second explanation might be the presence of a referral bias, with only fitter and better-nourished CKD patients being referred for nephrology care [3,4].

In our setting, the main biochemical features in the old, old-old and oldest-old groups were modulated by CKD stage rather than age (Supplementary Tables S2–S5).

A second interesting finding, also in partial disagreement with the current literature, is that baseline spontaneous protein intake at referral to nephrology care was not found to be affected by CKD stage or comorbidity (Supplementary Tables S6 and S7) [6]. Once more, the characteristics of this well-nourished, overweight French population with CKD, whose dietary habits had not previously been assessed, may have offset differences otherwise present in settings with a lower prevalence of obesity.

A third relevant finding is the lack of significant impact of baseline protein intake on mortality and morbidity, which were, as expected, modulated by age and comorbidity. In this context, the persistence of patients in the system was high and about 50% of them were still in follow-up 2 years after referral (Supplementary Figures S5–S7). This held true even for the oldest-old patients. The fact that patients tend to have a reasonably long follow-up in nephrology care means that there is potentially sufficient time for nutritional management to exert benefits.

Our study, like most real-life clinical studies, has drawbacks and limitations.

The study analyzes data obtained in a nephrology setting, and, as previously mentioned, a referral bias may exist. In particular, it is possible that it is the fittest and best-nourished elderly patients that are being selectively addressed to nephrology care. Competitive mortality may be the reason why elderly patients referred to the nephrology unit are mainly well nourished and have a high protein intake. Thus, our figures may not correctly reflect the habits of the entire elderly CKD population, but only of those selectively referred to our care. However, the estimate of the proportion of the CKD population currently being seen in our units was recently reported, and our data suggest that under-referral is more common in patients in CKD stage 3 [3]. While this is relevant for the nephrology workload, the lack of differences in protein intake across CKD stages found in the present study suggests that, at least in our setting, the results are not affected by the referral characteristics across stages.

Independently from referral biases, considering the recently published KDOQI guidelines on nutritional management in CKD patients, our study suggests that regardless of age and comorbidity, patients referred to a nephrology unit would benefit from nutritional assessment and probably also, at least in cases at higher risk for disease progression, from reduced protein intake according to the KDIGO guidelines [5].

A second limit is due to the fact that nutritional assessment was performed in most, but not all patients, by the 7 days diet journal. However, since not all patients are compliant with this quite demanding evaluation, in cases in which the diet journal was not available, shorter records (at least 3 days), dietary recall or direct calculations based upon the menus delivered (for retirement homes and home meal delivery) were used. Indeed, we consider that, while heterogeneity may be a limitation, choosing only evaluations performed in patients compliant to the diet journal would have introduced a selection bias, and we believe that completeness is an added value of our study. Furthermore, the fact that our dietitians spend one hour with the patient during the initial evaluation assures that all the efforts to compensate the non-homogeneity of data supplied for the nutritional evaluation have been made.

As our study was undertaken recently, a further limit is that we do not have enough follow-up data to analyze the effects of the nutritional interventions proposed for individuals in different age groups. To try to address this issue, a prospective study was started in 2019.

In conclusion, our data demonstrate that most old-old and oldest-old CKD patients referred to nephrology care have a spontaneous protein intake that is higher than recommended, and that there may still be a need for dietary intervention aimed at protein normalization or restriction even in patients at high comorbidity or in advanced CKD stages. Our study supports an urgent need for improving nutritional care in the elderly, high comorbidity population with advanced CKD.

## Figures and Tables

**Table 1:** Baseline data: the cohort followed-up at unit dedicated to advanced CKD (UIRAV) according to age groups (13 November 2017 to 31 December 2020).

	Age Groups					
	<60	60–69	70–79	80–89	≥90	*p*-Values
*N* (all: 436)	62	74	106	140	54	
**Males/females**	35/27	51/23	75/31	79/61	18/36	**<0.001**
Creatinine (mg/dL), median (IQR)	2.76 (2.39)	2.58 (1.32)	2.20 (1.09)	2.02 (1.02)	1.86 (1.08)	**<0.001**
eGFR CKD-EPI (mL/min/1.73 m^2^), median (IQR)	22 (23)	23 (19)	26 (15)	26 (15)	26 (17)	0.746
Proteinuria (g/24 h), *n* (%)						**<0.001**
<0.3	13 (21.0%)	16 (27.1%)	38 (47.5%)	56 (54.3%)	28 (73.6%)	
0.3–1	12 (19.3%)	9 (15.3%)	13 (16.3%)	25 (24.3%)	5 (13.2%)	
≥1	26 (41.9%)	34 (57.6%)	29 (36.2%)	22 (21.4%)	5 (13.2%)	
Stages, *n* (%)						0.419
3A	10 (16.1%)	6 (8.1%)	11 (10.4%)	10 (7.1%)	5 (9.2%)	
3B	16 (25.8%)	21 (28.4%)	32 (30.2%)	42 (30.0%)	17 (31.5%)	
4	20 (32.3%)	33 (44.6%)	45 (42.5%)	71 (50.7%)	21 (38.9%)	
5	16 (25.8%)	14 (18.9%)	18 (17.0%)	17 (12.2%)	11 (20.4%)	
Main diagnosis of kidney disease, *n* (%)						**<0.001**
ADPKD	5 (8.1%)	4 (5.4%)	1 (0.9%)	2 (1.4%)	1 (1.9%)	
DN	1 (1.6%)	0 (0%)	3 (2.9%)	1 (0.7%)	0 (0%)	
GN	6 (9.7%)	2 (2.7%)	1 (0.9%)	1 (0.7%)	2 (3.7%)	
Multifactorial	28 (45.2%)	40 (54.0%)	62 (58.5%)	63 (45.0%)	16 (29.6%)	
NAS or VN	5 (8.1%)	11 (14.9%)	25 (23.6%)	64 (45.7%)	31 (57.4%)	
Other	17 (27.4%)	17 (23.0%)	14 (13.2%)	9 (6.5%)	4 (7.4%)	

IQR: Interquartile range; ADPKD: Autosomal Dominant Polycystic Kidney Disease; DN diabetic nephropathy; GN: Glomerulonephritis; NAS: nephroangiosclerosis; VN: vascular nephropathy; eGFR: estimated glomerular filtration rate according to the Chronic Kidney Disease EPIdemiology collaboration formula. Four patients who alternated between CKD stages 2 and 3A, were considered CKD stage 3A. In bold, significant differences.

**Table 2:** Comorbidities, Malnutrition-Inflammation Score and Subjective Global Assessment Score in different age groups of CKD stage 3–5 patients.

	Age Groups					
	<60	60–69	70–79	80–89	≥90	*p*-Values
*N* (all: 436)	62	74	106	140	54	
**Males/females**	35/27	51/23	75/31	79/61	18/36	**<0.001**
CCI, median (IQR)	5 (3)	7 (4)	8 (2)	8 (3)	8 (1)	**<0.001**
MIS, median (IQR)	4 (4)	5 (4)	5 (4)	5 (3)	7 (4)	**<0.001**
SGA, *n* (%)						**<0.001**
A	57 (91.9%)	61 (82.4%)	94 (88.7%)	105 (75.5%)	23 (42.6%)	
B	3 (4.8%)	12 (16.2%)	11 (10.4%)	31 (22.3%)	29 (53.7%)	
C	1 (1.6%)	1 (1.4%)	1 (0.9%)	3 (2.2%)	2 (3.7%)	
Diabetes, *n* (%)	21 (33.9%)	41 (55.4%)	59 (55.7%)	58 (41.4%)	13 (24.1%)	**<0.001**
Ischemic heart disease, *n* (%)	10 (16.1%)	24 (32.4%)	37 (34.9%)	64 (45.7%)	22 (40.7%)	**0.004**
Neoplasia, *n* (%)	5 (8.1%)	13 (17.6%)	17 (16.0%)	31 (22.1%)	7 (13.0%)	0.201
BMI (kg/m^2^), median (IQR)	28.0 (9.8)	29.1 (10.2)	29.6 (8.8)	27.8 (5.5)	25.7 (6.2)	**0.002**
BMI classifications, *n* (%)						**<0.001**
<20 kg/m^2^	5 (8.1%)	5 (6.8%)	2 (1.9%)	4 (2.9%)	5 (9.6%)	
20–25 kg/m^2^	15 (24.2%)	19 (25.6%)	20 (18.9%)	25 (17.9%)	18 (34.6%)	
25–30 kg/m^2^	17 (27.4%)	18 (24.3%)	34 (32.1%)	67 (47.9%)	18 (34.6%)	
30–35 kg/m^2^	12 (19.4%)	17 (23.0%)	26 (24.5%)	33 (23.5%)	8 (15.4%)	
≥35 kg/m^2^	13 (21.0%)	15 (20.3%)	24 (22.6%)	11 (7.8%)	3 (5.8%)	

IQR: interquartile range; BMI: body mass index; CCI: Charlson Comorbidity Index; MIS: Malnutrition-Inflammation Score; SGA: Subjective Global Assessment. MIS and SGA are routinely assessed in all patients. However, 4 patients did not have complete MIS, SGA and BMI data (2 patients with missing MIS and SGA and 2 patients with missing BMI), mainly because the three indexes are calculated on the basis of an extensive clinical and biochemical evaluation, usually performed in the day hospital, after a first nephrology consultation. The missing data therefore regard either patients in the evaluation phase, or those who came to only one consultation. Four patients who alternated between CKD stages 2 and 3A, were considered CKD stage 3A. In bold, significant differences.

**Table 3:** Protein intake in different age groups of CKD stage 3–5 patients.

	Age Groups						
	<60	50–59	60–69	70–79	80–89	≥90	*p*-Values
*N* (all: 436)	62	39	74	106	140	54	
Creatinine at referral (mg/dL), median (IQR)	2.76 (2.39)	2.50 (2.23)	2.58 (1.32)	2.20 (1.09)	2.02 (1.02)	1.86 (1.08)	**<0.001**
eGFR (mL/min/1.73 m^2^), median (IQR)	22 (23)	24 (22)	23 (19)	26 (15)	26 (15)	26 (17)	0.746
Protein intake at baseline (g/kg/24 h), median (IQR)	1.20 (0.20)	1.20 (0.18)	1.10 (0.20)	1.00 (0.30)	1.00 (0.30)	1.00 (0.30)	**<0.001**
Details of protein intake at baseline: *n* (%)							0.227
≤0.8 g/kg/day	2 (3.2%)	1 (2.6%)	5 (7.0%)	9 (9.2%)	14 (10.4%)	6 (11.8%)	
0.81–1.19 g/kg/day	22 (35.5%)	16 (42.1%)	38 (52.8%)	54 (55.1%)	75 (55.5%)	28 (54.9%)	
≥1.2 g/kg/day	33 (53.2%)	21 (55.3%)	29 (40.2%)	35 (35.7%)	46 (34.1%)	17 (33.3%)	

IQR: interquartile range; eGFR: estimated glomerular filtration rate according to the CKD-EPI formula. In bold, significant differences.

**Table 4:** Cox regression analysis of survival according to baseline diet and CCI.

		CI 95%			
	Hazard Ratio	Lower	Higher		*p*-Value
Protein intake at referral ≥ 0.8 g/kg/day	0.832	0.383	1.809	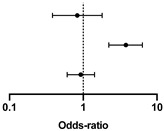	0.642
CCI dichotomized at median (≥8)	3.788	2.226	6.352		**<0.001**
Gender (female/male)	0.928	0.605	1.424		0.733

Concordance c-index: 0.65 (±0.029_SE_); AIC: 981.6; CCI: Charlson Comorbidity Index. In bold, significant differences.

**Table 5:** Cox regression analysis of survival according to baseline diet and age.

		CI 95%			
	Hazard Ratio	Lower	Higher		*p*-Value
Protein intake at referral ≥0.8 g/kg/day	0.876	0.401	1.913	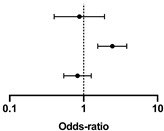	0.740
Age dichotomized at median (≥78 years old)	2.445	1.560	3.830		**<0.001**
Gender (female/male)	0.826	0.538	1.270		0.384

Concordance c-index: 0.62 (±0.028_SE_); AIC: 996.8. In bold, significant differences.

## Data Availability

The data presented in this study are available on request from the corresponding author.

## Supplementary Materials

The following are available online at https://www.mdpi.com/article/10.3390/nu13124371/s1, Table S1. Biochemical data according to different age groups in CKD stage 3–5 patients; Table S2. Biochemical data across stages: age 60–69; Table S3. Biochemical data across stages: age 70–79; Table S4. Biochemical data across stages: age 80–89; Table S5. Biochemical data across stages: age ≥90; Table S6. Distribution of protein intake across stages and ages; Table S7. Distribution of protein intake across CCI groups and ages; Table S8. Cox regression analysis of survival according to baseline diet and CCI; Table S9. Cox regression analysis of survival according to baseline diet and age; Figure S1. Visual aid for the serving size currently employed in our service to integrate the 7-day food diary and the interview with the dietician; Figure S2. Charlson Comorbidity Index distribution according to age groups; Figure S3. Malnutrition-Inflammation Score distribution according to age groups; Figure S4. Subjective Global Assessment distribution according to age groups; Figure S5. Renal survival in patients followed up at UIRAV, according to age at referral; Figure S6. Total drop out (death, dialysis start, or loss to follow-up) in patients at UIRAV, according to age at referral; Figure S7. Survival in patients followed up at UIRAV, according to age at referral.
