# Supplementary material for: Spontaneously Low Protein Intake in Elderly CKD Patients: Myth or Reality? Analysis of Baseline Protein Intake in a Large Cohort of Patients with Advanced CKD
Source: Nutrients. 2021 Dec 6;13(12):4371. doi: 10.3390/nu13124371 (PMC8707092; doi:10.3390/nu13124371)
Supplement: Supplementary file 1 [file nutrients-13-04371-s001.zip › supplementary material.pdf]

Supplementary material

Table S1. Biochemical data according to different age groups in CKD stage 3-5 patients.

	Age groups					
	<60	60-69	70-79	80-89	≥90	P-values
N (all: 436)	62	74	106	140	54	
Creatinine (mg/dL), median (IQR)	2.76 (2.39)	2.58 (1.32)	2.20 (1.09)	2.02 (1.02)	1.86 (1.08)	**<0.001**
eGFR CKD-EPI (mL/min/1.73m^2), median (IQR)	22 (23)	23 (19)	26 (15)	26 (15)	26 (17)	0.746
Proteinuria (g/24h), n (%)						
<0.3	13 (21.0%)	16 (27.1%)	38 (47.5%)	56 (54.4%)	28 (73.7%)	**<0.001**
0.3 - 1	12 (19.4%)	9 (15.3%)	13 (16.3%)	25 (24.3%)	5 (13.2%)	
≥1	26 (41.9%)	34 (57.6%)	29 (36.3%)	22 (21.4%)	5 (13.2%)	
Albumin (g/dL), median (IQR)	3.6 (0.8)	3.7 (0.6)	3.8 (0.5)	3.6 (0.5)	3.5 (0.3)	**0.009**
Albumin (g/dL) < 3.5, n (%)	25 (40.3%)	29 (39.2%)	23 (21.7%)	42 (30.0%)	21 (38.9%)	0.055
Albumin (g/dL) < 3, n (%)	7 (11.3%)	5 (6.8%)	8 (7.5%)	7 (5.0%)	3 (5.6%)	0.416
Total serum proteins (g/L), median (IQR)	72 (7)	74 (8)	73 (9)	72 (8)	72 (8)	0.466
CRP (mg/L), median (IQR)	4 (4)	5 (11)	4 (5)	4 (7)	5 (9)	0.304
PTH (ng/L), median (IQR)	124 (200)	94 (116)	107 (104)	85 (74)	76 (56)	**0.027**
BUN (mg/dL), median (IQR)	42.3 (28.5)	42.0 (29.0)	39.5 (28.1)	42.6 (22.8)	41.9 (22.8)	0.901
Bicarbonate (mmol/L), median (IQR)	22 (5)	23 (4)	24 (5)	24 (5)	24 (4)	**<0.001**
Hemoglobin (g/dL), median (IQR)	11.7 (2.8)	11.7 (3.1)	11.8 (2.7)	11.6 (2.3)	11.5 (2.0)	0.881
Hemoglobin (g/dL) < 10, n (%)	10 (16.1%)	16 (21.6%)	15 (14.3%)	20 (14.3%)	8 (14.8%)	0.761
Ferritin (µg/L), median (IQR)	116 (222)	174 (210)	187 (222)	158 (177)	180 (183)	0.054
Transferrin (g/L), median (IQR)	2.22 (0.59)	2.30 (0.61)	2.20 (0.57)	2.28 (0.58)	2.22 (0.41)	0.436
Uric acid (µmol/L), median (IQR)	426 (195)	443 (190)	434 (159)	462 (193)	441 (146)	0.158
Total cholesterol (mmol/L), median (IQR)	4.61 (2.05)	4.87 (1.92)	4.32 (1.33)	4.46 (1.99)	5.08 (2.10)	**0.026**
Vitamin D (ng/mL), median (IQR)	23 (14)	28 (25)	27 (20)	30 (17)	32 (20)	**0.027**
Sodium (mmol/L), median (IQR)	141 (3)	140 (4)	140 (4)	141 (4)	140 (4)	0.779
Potassium (mmol/L), median (IQR)	4.5 (0.7)	4.3 (0.6)	4.2 (0.7)	4.3 (0.6)	4.3 (0.6)	**0.0039**
Calcium (mmol/L), median (IQR)	2.33 (0.14)	2.35 (0.19)	2.34 (0.16)	2.34 (0.17)	2.33 (0.14)	0.361
Phosphate (mmol/L), median (IQR)	1.24 (0.49)	1.09 (0.35)	1.05 (0.31)	1.10 (0.28)	1.14 (0.32)	**0.043**
Diabetes, n (%)	21 (33.9%)	41 (55.4%)	59 (55.7%)	58 (41.4%)	13 (24.1%)	**<0.001**
HbA1c (%) *, median (IQR)	7.3 (1.3)	7.0 (2.0)	6.9 (1.6)	6.8 (1.5%)	7.2 (0.8)	0.996

IQR: interquartile range; eGFR: estimated glomerular filtration rate according to the CKD-EPI formula; PTH: parathyroid hormone; BUN: blood urea nitrogen. * only for patients with diabetes, 95% available data. In bold, significant differences.

Table S2. Biochemical data across stages: age 60-69.

60-69 years (n=74)					
	CKD Stages				
	3A	3B	4	5	P-values
N	6	21	33	14	
Creatinine (mg/L), median (IQR)	1.33 (0.06)	1.92 (0.28)	2.89 (0.61)	5.21 (2.34)	**<0.001**
eGFR EPI (mL/min/1.73m^2), median (IQR)	55 (6)	35 (4)	20 (5)	10 (5)	**<0.001**
Charlson index, median (IQR)	8 (4)	6 (4)	8 (3)	7 (3)	0.085
MIS, median (IQR)	7 (2)	3 (3)	5 (4)	6 (4)	0.382
SGA, n (%)					
A	6 (100%)	18 (85.7%)	25 (75.8%)	12 (85.7%)	0.877
B	0	3 (14.3%)	7 (21.2%)	2 (14.3%)	
C	0	0	1 (3.0%)	0	
Diabetes, n (%)	3 (50.0%)	8 (38.1%)	22 (66.7%)	8 (57.1%)	0.225
Cardiopathy, n (%)	5 (83.3%)	5 (23.8%)	10 (30.3%)	4 (28.6%)	0.060
Neoplasia, n (%)	1 (16.7%)	4 (19.0%)	8 (24.2%)	0 (0%)	0.221
BMI (kg.m^{-2}), median (IQR)	25.9 (10.1)	29.4 (9.0)	29.2 (8.0)	27.2 (10.8)	0.714
Albumin (g/dL), median (IQR)	3.8 (0.3)	3.7 (0.8)	3.5 (0.6)	3.6 (0.5)	0.298
Total serum proteins (g/L), median (IQR)	74 (2)	75 (8)	74 (6)	73 (12)	0.779
CRP (mg/L), median (IQR)	19 (38)	5 (8)	4 (7)	6 (14)	0.381
PTH (ng/L), median (IQR)	70 (20)	48 (53)	101 (107)	285 (659)	**<0.001**
BUN (mg/dL), median (IQR)	28.1 (4.3)	31.9 (19.3)	47.4 (23.0)	73.0 (46.0)	**<0.001**
Bicarbonate (mmol/L), median (IQR)	25 (1)	23 (3)	23 (5)	21 (4)	0.134
Hemoglobin (g/dL), median (IQR)	13.0 (1.1)	12.5 (2.7)	11.3 (2.5)	10.1 (2.7)	**0.032**
Ferritin (µg/L), median (IQR)	177 (147)	164 (247)	140 (136)	239 (197)	0.644
Transferrin (g/L), median (IQR)	2.42 (0.40)	2.41 (0.54)	2.22 (0.63)	2.03 (0.47)	0.182
Uric acid (µmol/L), median (IQR)	384 (111)	427 (179)	481 (218)	442 (138)	0.290
Total cholesterol (mmol/L), median (IQR)	5.00 (1.58)	5.37 (1.28)	4.71 (1.96)	3.99 (2.12)	0.214
Vit D (ng/mL), median (IQR)	26 (15)	32 (26)	29 (26)	27 (19)	0.771
Sodium (mmol/L), median (IQR)	141 (2)	139 (3)	140 (4)	140 (4)	0.808
Potassium (mmol/L), median (IQR)	4.3 (0.5)	3.9 (0.5)	4.3 (0.4)	4.3 (1.1)	0.053
Calcium (mmol/L), median (IQR)	2.29 (0.13)	2.42 (0.13)	2.35 (0.18)	2.25 (0.15)	0.109
Phosphate (mmol/L), median (IQR)	0.89 (0.19)	0.96 (0.18)	1.16 (0.28)	1.40 (0.26)	**<0.001**
Diabetes, n (%)	3 (50.0%)	8 (38.1%)	22 (66.7%)	8 (57.1%)	0.225
HbA1c (%) *, median (IQR)	8.1 (1.8)	8.0 (1.7)	6.9 (1.5)	5.9 (0.6)	**0.050**

IQR: interquartile range; eGFR: estimated glomerular filtration rate according to the CKD-EPI formula; MIS: Malnutrition-Inflammation index; SGA: Subjective Global Assessment; BMI: Body Mass Index; PTH: parathyroid hormone; BUN: blood urea nitrogen. * only for those with diabetes in this group, with 92% available data (for CKD stages 3A, 3B, 4 and 5: 3,8,20,7 respectively). In bold, significant differences.

Table S3. Biochemical data across stages: age 70-79.

**70-79 years old (n=106)**					
	CKD Stages				
	3A	3B	4	5	P-values
N	11	32	45	18	
Creatinine (mg/L), median (IQR)	1.25 (0.29)	1.82 (0.34)	2.58 (0.57)	4.58 (1.59)	**<0.001**
eGFR EPI (mL/min/1.73m^2), median (IQR)	47 (8)	35 (6)	23 (5)	11 (5)	**<0.001**
Charlson index, median (IQR)	6 (4)	8 (2)	8 (2)	8 (3)	**<0.001**
MIS, median (IQR)	5 (5)	4 (3)	6 (3)	5 (5)	0.112
SGA, n (%)					
A	8 (72.7%)	31 (96.9%)	41 (91.1%)	14 (77.8%)	0.053
B	3 (27.3%)	1 (3.1%)	3 (6.7%)	4 (22.2%)	
C	0 (0%)	0 (0%)	1 (2.2%)	0 (0%)	
Diabetes, n (%)	4 (36.4%)	13 (40.6%)	31 (68.9%)	11 (61.1%)	**0.045**
Cardiopathy, n (%)	2 (18.2%)	10 (31.3%)	19 (42.2%)	6 (33.3%)	0.489
Neoplasia, n (%)	2 (18.2%)	3 (9.4%)	7 (15.6%)	5 (27.8%)	0.367
BMI (kg.m^{-2}), median (IQR)	27.3 (3.8)	29.5 (6.2)	30.5 (9.7)	29.4 (11.8)	0.444
Albumin (g/dL), median (IQR)	3.9 (0.3)	3.9 (0.3)	3.7 (0.5)	3.8 (0.8)	0.066
Total serum proteins (g/L), median (IQR)	74 (7)	75 (8)	72 (10)	73 (7)	0.485
CRP (mg/L), median (IQR)	4 (20)	4 (3)	4 (5)	8 (16)	0.278
PTH (ng/L), median (IQR)	72 (25)	56 (48)	125 (91)	257 (148)	**<0.001**
BUN (mg/dL), median (IQR)	24.4 (4.9)	32.2 (11.9)	50.4 (23.1)	64.4 (27.2)	**<0.001**
Bicarbonate (mmol/L), median (IQR)	26 (3)	24 (4)	24 (5)	22 (4)	**0.006**
Hemoglobin (g/dL), median (IQR)	12.2 (3.2)	13.1 (2.6)	11.6 (2.2)	11.0 (1.6)	**0.002**
Ferritin (μg/L), median (IQR)	171 (226)	196 (223)	211 (196)	175 (396)	0.886
Transferrin (g/L), median (IQR)	2.19 (0.11)	2.36 (0.67)	2.19 (0.68)	2.20 (0.39)	**0.030**
Uric acid (μmol/L), median (IQR)	434 (107)	406 (86)	439 (188)	468 (168)	0.587
Total cholesterol (mmol/L), median (IQR)	4.82 (1.32)	4.67 (1.56)	4.00 (1.18)	4.01 (1.53)	0.223
Vit D (ng/mL), median (IQR)	24 (28)	25 (19)	27 (21)	26 (16)	0.803
Sodium (mmol/L), median (IQR)	139 (4)	139 (4)	141 (4)	141 (2)	0.061
Potassium (mmol/L), median (IQR)	4.2 (0.8)	4.2 (0.4)	4.2 (0.8)	4.5 (0.7)	0.160
Calcium (mmol/L), median (IQR)	2.32 (0.09)	2.41 (0.11)	2.32 (0.18)	2.29 (0.13)	**0.007**
Phosphate (mmol/L), median (IQR)	0.96 (0.16)	0.99 (0.20)	1.06 (0.31)	1.52 (0.50)	**<0.001**
Diabetes, n (%)	4 (36.4%)	13 (40.6%)	31 (68.9%)	11 (61.1%)	**0.045**
HbA1c (%) *, median (IQR)	6.4 (0.4)	6.9 (1.4)	7.2 (1.7)	6.8 (1.9)	**0.205**

IQR: interquartile range; eGFR: estimated glomerular filtration rate according to the CKD-EPI formula; MIS: Malnutrition-Inflammation index; SGA: Subjective Global Assessment; BMI: Body Mass Index; PTH: parathyroid hormone; BUN: blood urea nitrogen. * only for those with diabetes in this group, with 98% available data (for CKD stages 3A, 3B, 4 and 5: 4, 13, 30, 11 respectively). In bold, significant differences.

Table S4. Biochemical data across stages: age 80-89.

80-89 years old (n=138)					
	CKD Stages				
	3A	3B	4	5	P-values
N	10	42	71	17	
Creatinine (mg/L), median (IQR)	1.08 (0.28)	1.68 (0.30)	2.27 (0.69)	3.85 (1.57)	**<0.001**
eGFR EPI (mL/min/1.73m^2), median (IQR)	51 (4)	35 (5)	23 (8)	10 (5)	**<0.001**
Charlson index, median (IQR)	7 (1)	8 (3)	9 (2)	8 (3)	**0.027**
MIS, median (IQR)	5 (3)	6 (4)	5 (4)	6 (6)	0.218
SGA, n (%)					
A	8 (80.0%)	31 (75.6%)	56 (78.8%)	10 (58.8%)	0.192
B	2 (20.0%)	9 (22.0%)	15 (21.2%)	5 (29.4%)	
C	0 (0%)	1 (2.4%)	0 (0%)	2 (11.8%)	
Diabetes, n (%)	2 (20.0%)	15 (35.7%)	35 (49.3%)	6 (35.3%)	0.235
Cardiopathy, n (%)	5 (50.0%)	17 (40.5%)	34 (47.9%)	8 (47.1%)	0.873
Neoplasia, n (%)	0 (0%)	13 (31.0%)	16 (22.5%)	2 (11.8%)	0.130
BMI (kg.m^{-2}), median (IQR)	30.2 (7.5)	26.6 (4.2)	28.5 (5.5)	26.1 (4.4)	0.175
Albumin (g/dL), median (IQR)	3.8 (0.4)	3.8 (0.6)	3.6 (0.4)	3.4 (0.5)	**0.032**
Total serum proteins (g/L), median (IQR)	69 (7)	71 (8)	74 (8)	72 (10)	0.146
CRP (mg/L), median (IQR)	4 (3)	5 (9)	3 (6)	5 (7)	0.702
PTH (ng/L), median (IQR)	49 (36)	78 (53)	91 (74)	172 (135)	**<0.001**
BUN (mg/dL), median (IQR)	27.9 (14.8)	31.4 (15.1)	45.8 (19.3)	57.0 (29.7)	**<0.001**
Bicarbonate (mmol/L), median (IQR)	26 (5)	25 (5)	24 (4)	20 (4)	**<0.001**
Hemoglobin (g/dL), median (IQR)	12.2 (1.5)	11.8 (2.4)	11.6 (2.0)	9.9 (1.8)	**<0.001**
Ferritin (µg/L), median (IQR)	163 (190)	161 (188)	144 (148)	180 (191)	0.414
Transferrin (g/L), median (IQR)	2.23 (0.62)	2.15 (0.41)	2.37 (0.49)	2.14 (0.80)	0.180
Uric acid (µmol/L), median (IQR)	437 (84)	442 (154)	518 (208)	485 (184)	0.081
Total cholesterol (mmol/L), median (IQR)	5.10 (1.68)	4.34 (1.61)	4.34 (2.24)	4.87 (1.45)	0.096
Vit D (ng/mL), median (IQR)	37 (15)	27 (17)	32 (16)	31 (23)	0.727
Sodium (mmol/L), median (IQR)	141 (2)	141 (4)	140 (4)	139 (6)	0.147
Potassium (mmol/L), median (IQR)	4.2 (0.6)	4.3 (0.7)	4.3 (0.5)	4.3 (0.6)	0.886
Calcium (mmol/L), median (IQR)	2.28 (0.22)	2.36 (0.16)	2.34 (0.15)	2.34 (0.26)	0.165
Phosphate (mmol/L), median (IQR)	1.02 (0.15)	1.02 (0.24)	1.11 (0.26)	1.43 (0.35)	**<0.001**
Diabetes, n (%)	2 (20.0%)	15 (35.7%)	35 (49.3%)	6 (35.3%)	0.235
HbA1c (%) *, median (IQR)	7.1 (0.5)	7.1 (1.5)	6.9 (1.8)	6.4 (1.4)	0.692

IQR: interquartile range; eGFR: estimated glomerular filtration rate according to the CKD-EPI formula; MIS: Malnutrition-Inflammation index; SGA: Subjective Global Assessment; BMI: Body Mass Index; PTH: parathyroid hormone; BUN: blood urea nitrogen. * only for those with diabetes in this group, with 98% available data (for CKD stages 3A, 3B, 4 and 5: 2, 14, 35, 6, respectively). In bold, significant differences.

Table S5. Biochemical data across stages: age ≥90.

**≥90 years old (n=53)**					
	CKD Stages				
	3A	3B	4	5	P-values
N	5	17	21	11	
Creatinine (mg/L), median (IQR)	1.07 (0.21)	1.44 (0.22)	2.11 (0.66)	4.06 (0.85)	**<0.001**
eGFR EPI (mL/min/1.73m^2), median (IQR)	46 (4)	35 (7)	22 (7)	10 (3)	**<0.001**
Charlson index, median (IQR)	8 (1)	9 (1)	8 (2)	9 (2)	0.741
MIS, median (IQR)	6 (1)	5 (2)	7 (4)	9 (4)	0.349
SGA, n (%)					
A	2 (40%)	8 (47.1%)	9 (42.9%)	4 (36.4%)	0.481
B	3 (60%)	9 (52.9%)	12 (57.1%)	5 (45.4%)	
C	0 (0%)	0 (0%)	0 (0%)	2 (18.2%)	
Diabetes, n (%)	1 (20%)	5 (29%)	4 (19%)	3 (27%)	0.900
Cardiopathy, n (%)	4 (80%)	5 (29%)	7 (33%)	6 (55%)	0.160
Neoplasia, n (%)	0 (0%)	2 (12%)	3 (14%)	2 (18%)	0.999
BMI (kg.m^{-2}), median (IQR)	24.9 (8.1)	27.8 (4.9)	24.0 (4.4)	27.8 (5.7)	0.394
Albumin (g/dL), median (IQR)	3.7 (0.0)	3.6 (0.2)	3.4 (0.5)	3.5 (0.2)	0.143
Total serum proteins (g/L), median (IQR)	71 (3)	73 (8)	72 (9)	71 (5)	0.705
CRP (mg/L), median (IQR)	3 (3)	3 (5)	8 (11)	10 (10)	0.310
PTH (ng/L), median (IQR)	64 (63)	83 (53)	56 (56)	162 (180)	0.051
BUN (mg/dL), median (IQR)	28.3 (7.8)	33.3 (13.0)	45.7 (10.1)	70.0 (40.8)	**0.009**
Bicarbonate (mmol/L), median (IQR)	24 (3)	25 (4)	24 (6)	22 (6)	0.140
Hemoglobin (g/dL), median (IQR)	12.8 (1.9)	12.4 (1.8)	11.2 (1.5)	10.5 (1.7)	**0.005**
Ferritin (µg/L), median (IQR)	135 (136)	158 (158)	231 (220)	176 (190)	0.795
Transferrin (g/L), median (IQR)	2.33 (0.18)	2.26 (0.21)	2.13 (0.41)	2.06 (1.00)	0.492
Uric acid (µmol/L), median (IQR)	459 (62)	414 (121)	458 (155)	500 (149)	0.718
Total cholesterol (mmol/L), median (IQR)	4.66 (1.04)	5.22 (1.65)	5.28 (2.10)	5.02 (2.79)	0.706
Vit D (ng/mL), median (IQR)	34 (11)	30 (18)	35 (22)	22 (30)	0.298
Sodium (mmol/L), median (IQR)	141 (3)	142 (5)	140 (4)	139 (5)	0.884
Potassium (mmol/L), median (IQR)	4.3 (0.0)	3.9 (0.6)	4.4 (0.2)	4.0 (1.3)	**0.048**
Calcium (mmol/L), median (IQR)	2.38 (0.12)	2.35 (0.15)	2.35 (0.16)	2.29 (0.15)	**0.021**
Phosphate (mmol/L), median (IQR)	0.98 (0.03)	1.06 (0.18)	1.14 (0.23)	1.49 (0.64)	**0.002**
Diabetes, n (%)	1 (20%)	5 (29%)	4 (19%)	3 (27%)	0.900
HbA1c (%) *, median (IQR)	7.43 (0.0)	7.17 (0.41)	6.53 (0.85)	7.35 (0.59)	-

IQR: interquartile range; eGFR: estimated glomerular filtration rate according to the CKD-EPI formula; MIS: Malnutrition-Inflammation index; SGA: Subjective Global Assessment; BMI: Body Mass Index; PTH: parathyroid hormone; BUN: blood urea nitrogen. * only for those with diabetes in this group, with 92% available data (for CKD stages 3A, 3B, 4 and 5: 1, 4, 4, 3, respectively). In bold, significant differences.

Table S6. Distribution of protein intake across stages and ages.

	Age												
	<60				60 - 79				≥80				
	Stage				Stage				Stage				
	3	4	5	p-value	3	4	5	p-value	3	4	5	p-value	overall
N	26	20	16		70	78	32		74	92	28		
Protein intake at referral, median (IQR)	1.20 (0.20)	1.20 (0.30)	1.20 (0.18)	0.968	1.10 (0.23)	1.10 (0.20)	1.00 (0.38)	0.203	1.00 (0.30)	1.00 (0.30)	1.00 (0.20)	0.912	0.786
Protein intake classifications, n (%)				0.352				0.515				0.746	
≤0.8 g/kg/d	0 (0.0%)	1 (5.9%)	1 (7.1%)		6 (9.0%)	4 (5.4%)	4 (13.8%)		8 (11.1%)	9 (10.2%)	3 (11.5%)		
0.81-0.19 g/kg/d	13 (50.0%)	5 (29.4%)	4 (28.6%)		38 (56.7%)	38 (51.4%)	16 (55.2%)		36 (50.0%)	53 (60.2%)	14 (53.9%)		
≥1.2 g/kg/d	13 (50.0%)	11 (64.7%)	9 (64.3%)		23 (34.3%)	32 (43.2%)	9 (31.0%)		28 (38.9%)	26 (29.6%)	9 (34.6%)		

IQR: interquartile range.

Table S7. Distribution of protein intake across CCI groups and ages.

	Age												
	<60				60 - 79				≥80				
	CCI				CCI				CCI				overall
	≤7	8 to 10	≥11	p-value	≤7	8 to 10	≥11	p-value	≤7	8 to 10	≥11	p-value	
N	53	9	0		84	79	17		60	106	28		
Protein intake at referral, median (IQR)	1.20 (0.19)	1.20 (0.20)	-	0.434	1.10 (0.20)	1.00 (0.30)	1.10 (0.20)	0.992	1.00 (0.20)	1.10 (0.30)	1.00 (0.35)	0.223	0.105
Protein intake classifications, n (%)				0.999				0.778				0.369	
≤0.8 g/kg/d	2 (4.0%)	0 (0%)	-		7 (8.5%)	5 (6.7%)	2 (15.4%)		5 (8.6%)	9 (8.9%)	6 (22.2%)		
0.81-0.19 g/kg/d	19 (38.0%)	3 (42.9%)			43 (52.5%)	43 (57.3%)	6 (46.1%)		34 (58.6%)	55 (54.5%)	14 (51.9%)		
≥1.2 g/kg/d	29 (58.0%)	4 (57.1%)	-		32 (39.0%)	27 (36.0%)	5 (38.5%)		19 (32.8%)	37 (36.6%)	7 (25.9%)		

IQR: interquartile range.

nutrients

MDPI

Table S8. Cox regression analysis of survival according to baseline diet and CCI .

	Hazard ratio	CI 95% Lower	CI 95% Higher	p-value
Protein intake at referral ≥1 g/kg/day	1.053	0.639	1.737	0.838
CCI dichotomized at median (≥8)	3.812	2.271	6.398	**<0.001**
Gender (female/male)	0.936	0.611	1.434	0.761

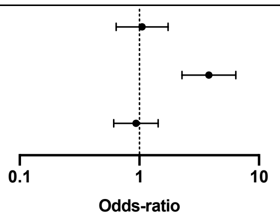

Concordance c-index: 0.65 (±0.029SE); AIC: 981.8; CCI: Charlson Comorbidity Index.

Table S9. Cox regression analysis of survival according to baseline diet and age.

	Hazard ratio	CI 95% Lower	CI 95% Higher	p-value
Protein intake at referral ≥1g/kg/day	1.007	0.611	1.660	0.979
Age dichotomized at median (≥78 years old)	2.458	1.570	3.850	**<0.001**
Gender (female/male)	0.832	0.543	1.276	0.400

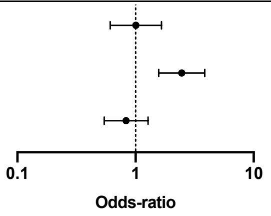

Concordance c-index: 0.61 (±0.028SE); AIC: 996.9. In bold, significant differences.

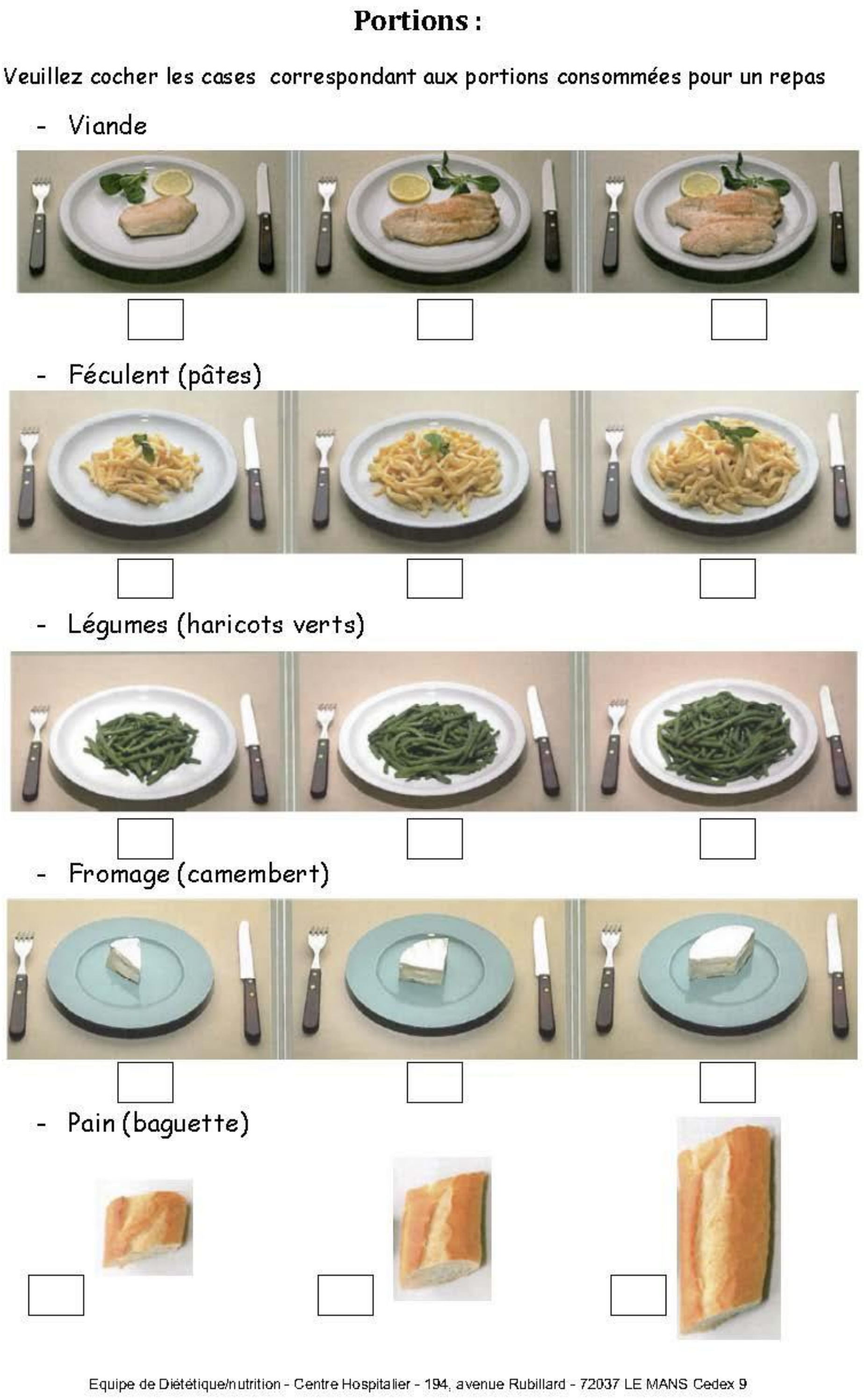

Figure S1. Visual aid for the serving size currently employed in our service to integrate the 7-day food diary and the interview with the dietician.

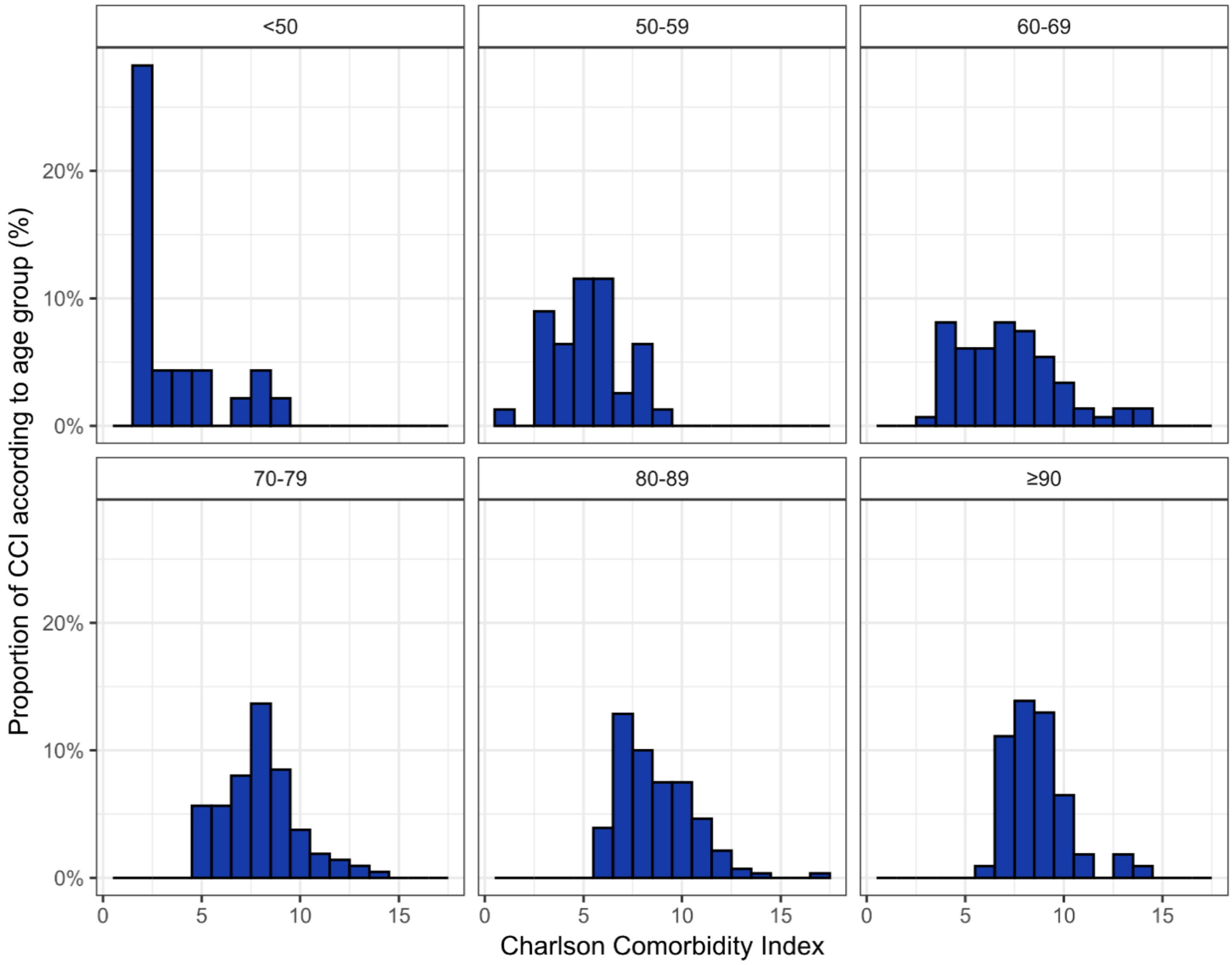

Figure S2. Charlson Comorbidity Index distribution according to age groups.

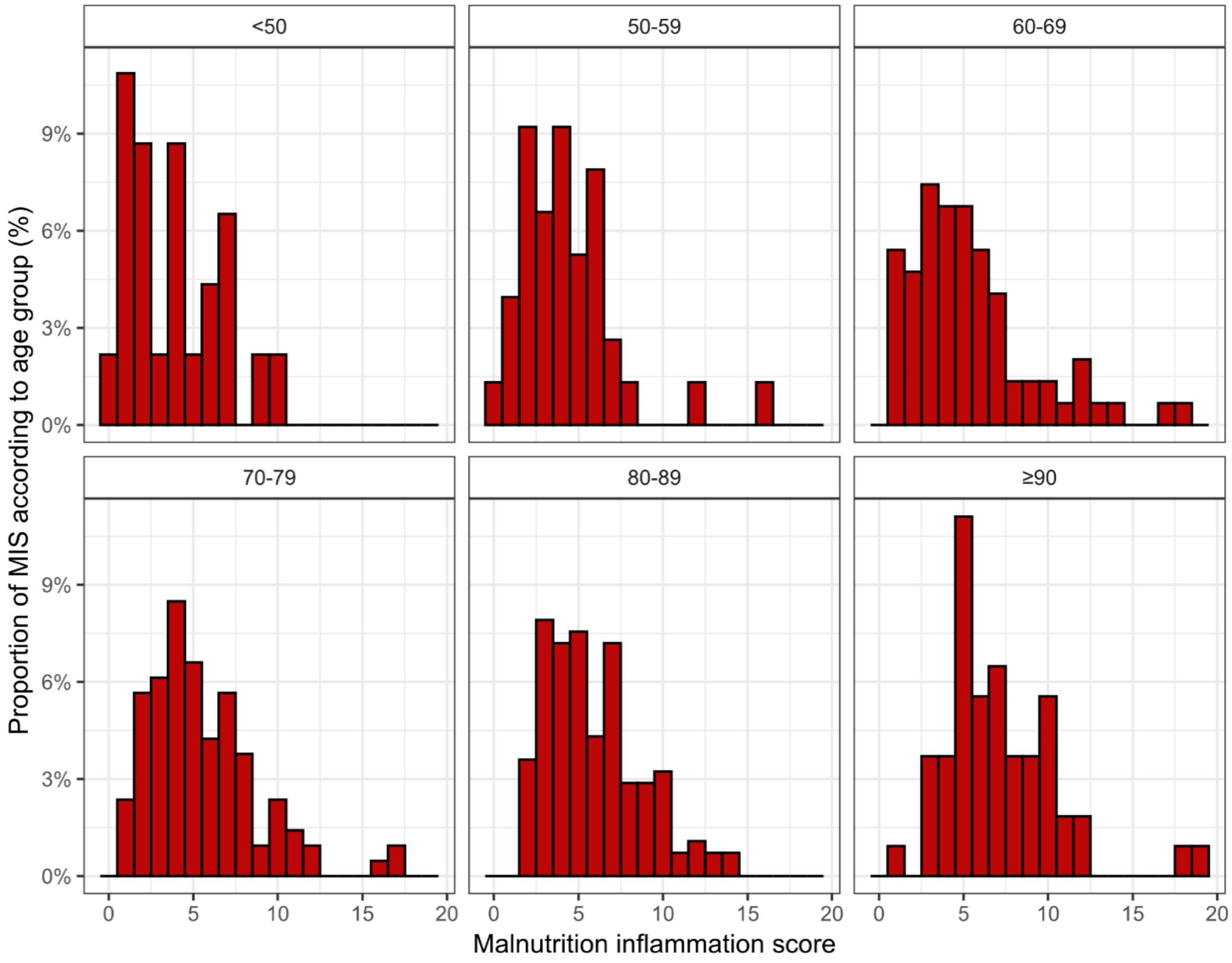

Figure S3. Malnutrition-Inflammation Score distribution according to age groups.

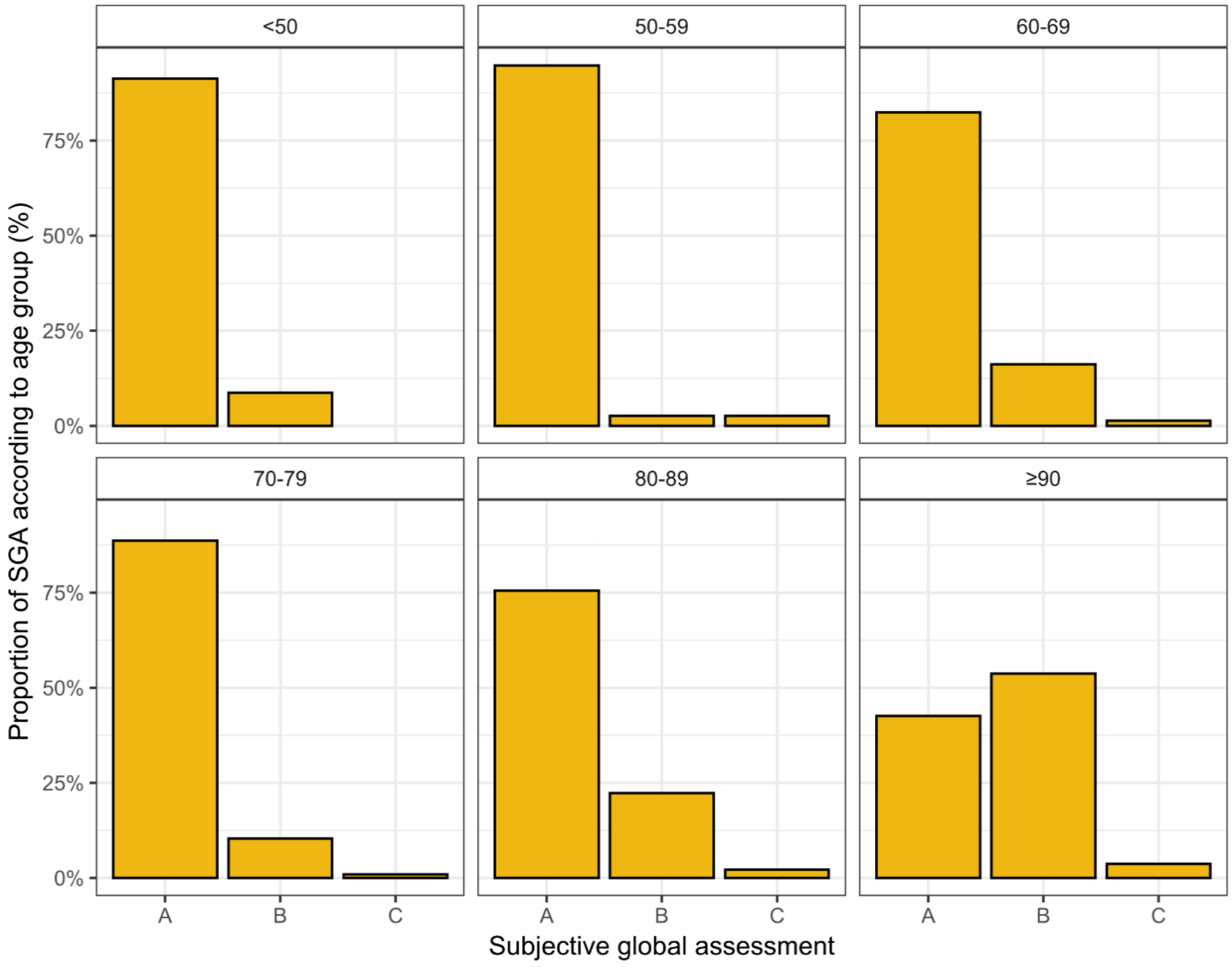

Figure S4. Subjective Global Assessment distribution according to age groups.

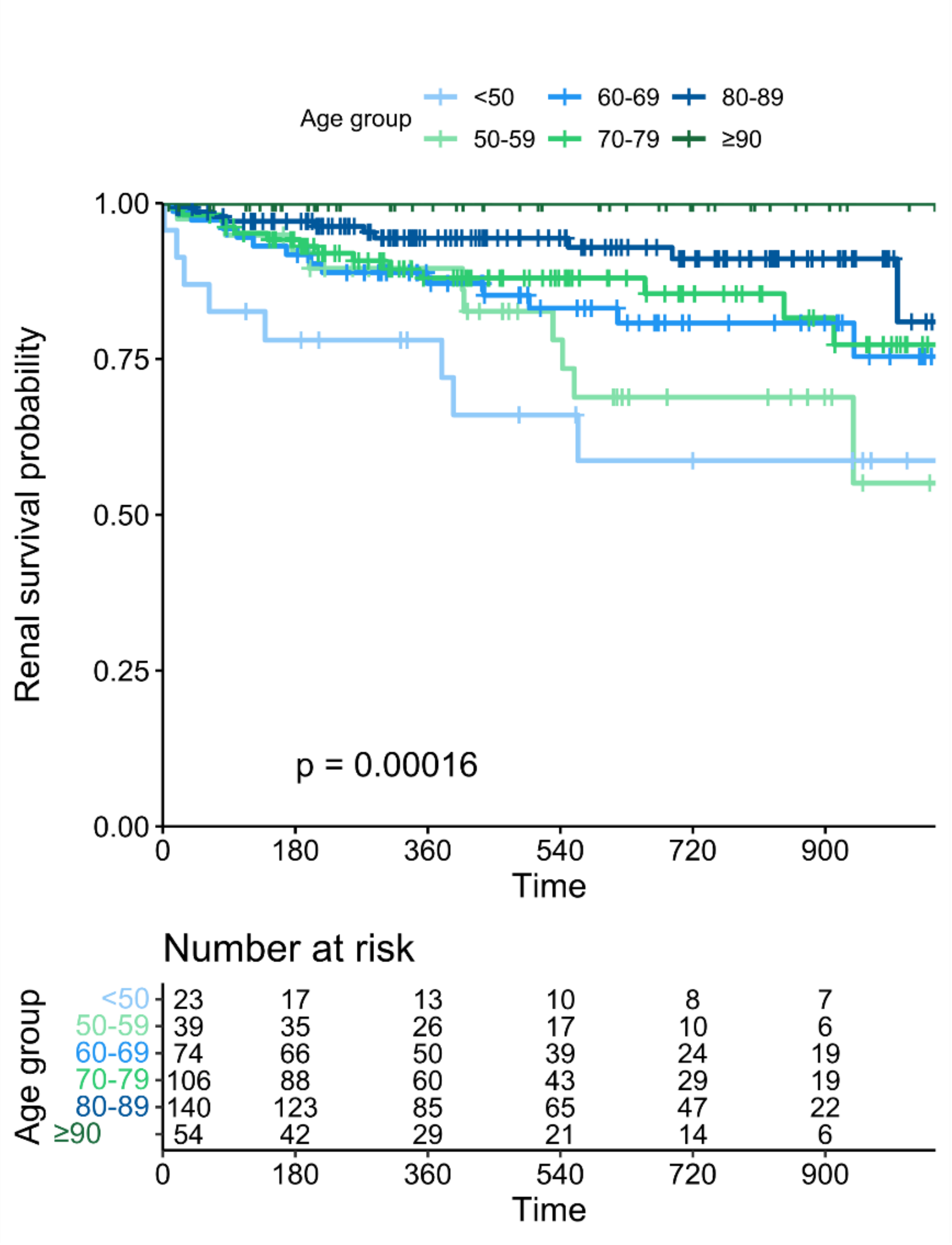

Figure S5. Renal survival in patients followed up at UIRAV, according to age at referral.

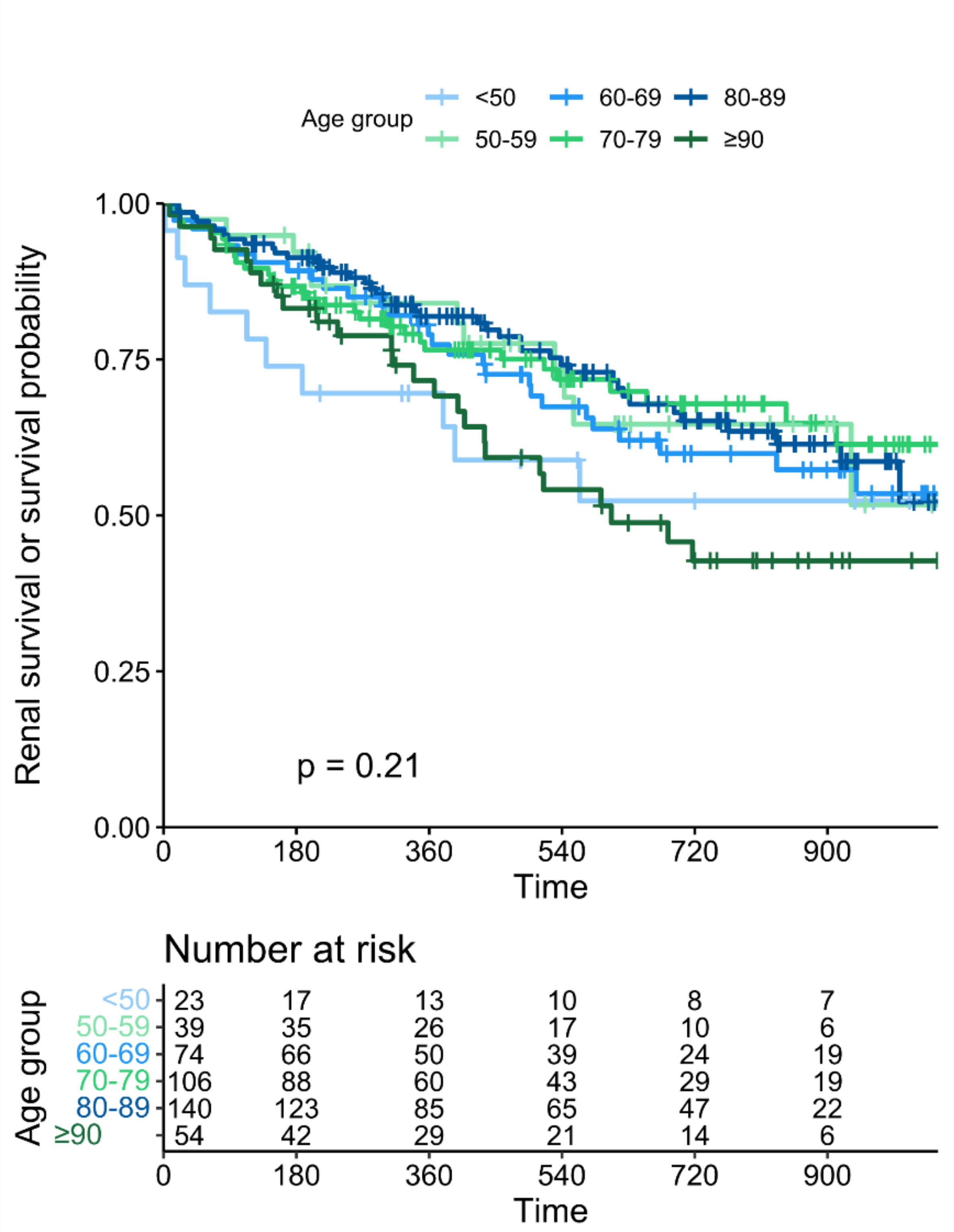

Figure S6. Total drop out (death, dialysis start, or loss to follow-up) in patients at UIRAV, according to age at referral.

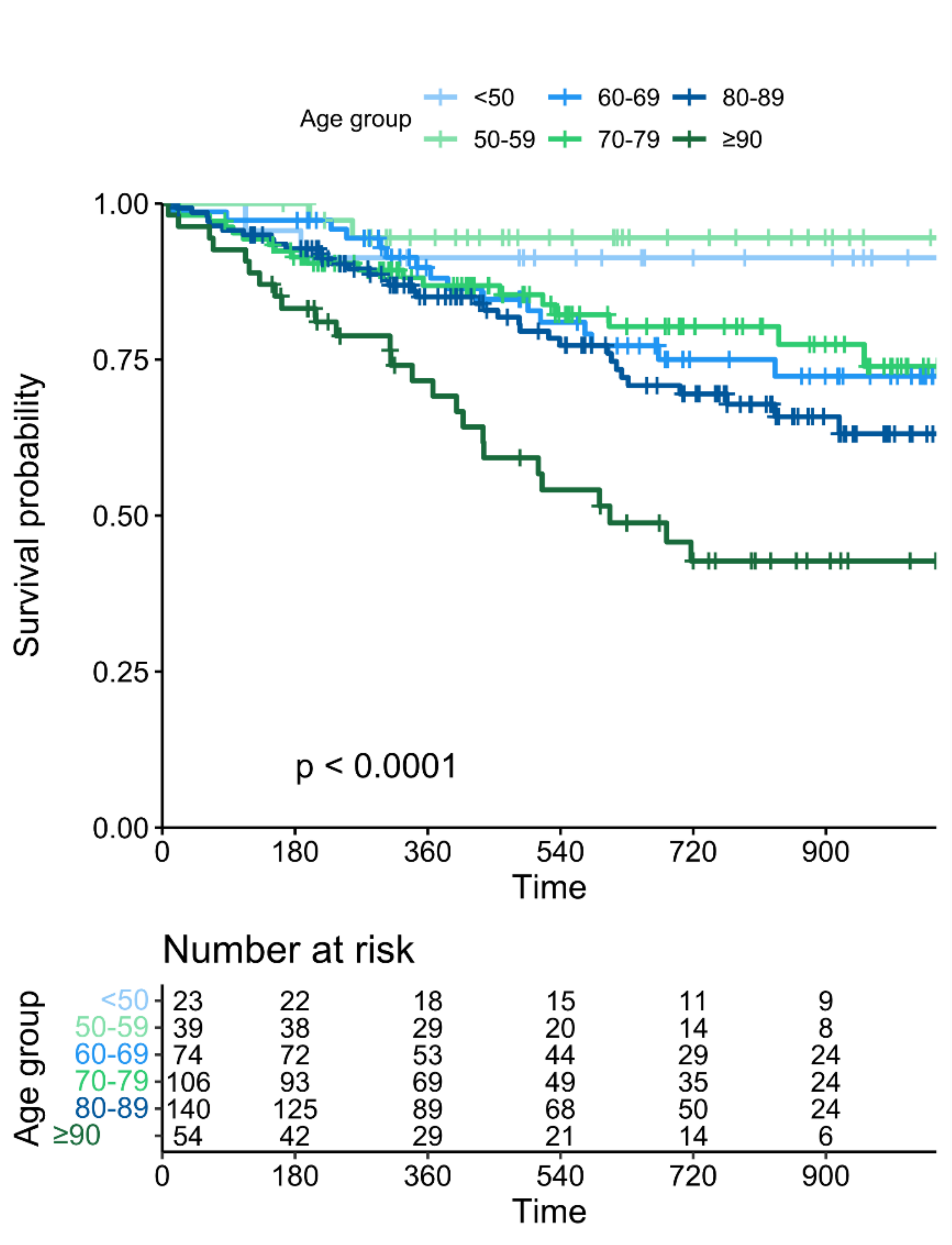

Figure S7. Survival in patients followed up at UIRAV, according to age at referral.
